# Botulinum Toxin for Pediatric Vocal Cord Dysfunction: A Case Series

**DOI:** 10.7759/cureus.96252

**Published:** 2025-11-06

**Authors:** Ahmed H Ali, German P Digoy

**Affiliations:** 1 Otolaryngology, Creighton University School of Medicine, Omaha, USA; 2 Pediatric Otolaryngology, Oklahoma State University Center for Health Sciences, Tulsa, USA

**Keywords:** botulinum toxin, laryngeal dystonia, paradoxical vocal fold motion, pediatric, tracheostomy, vocal cord dysfunction

## Abstract

Introduction

Vocal cord dysfunction (VCD) in children is a rare but challenging airway disorder characterized by inappropriate vocal fold adduction during inspiration. In pediatric populations, it is often associated with neurologic comorbidities or refractory symptoms that do not respond to conventional therapies such as voice therapy or medical management. Botulinum toxin (BTX) has emerged as a potential therapeutic option due to its ability to transiently weaken overactive laryngeal muscles while preserving essential airway and phonatory function. However, evidence on its efficacy and safety in children remains limited.

Methods

We conducted a retrospective case series of 10 pediatric patients with confirmed VCD treated with BTX at a tertiary pediatric otolaryngology center between 2021 and 2024. All patients underwent pre- and post-treatment laryngoscopy with exercise laryngoscopy when feasible. Swallowing function was assessed via fiberoptic endoscopic evaluation of swallowing (FEES) or modified barium swallow studies.

Results

Ten patients (six female patients and four male patients; ages 12 days to 17 years) met the inclusion criteria. Primary indications included refractory inspiratory stridor (n = 10), severe dysphagia (n = 6), and failed conservative management. Four patients had tracheostomies for severe VCD-related obstruction; all were ultimately decannulated, although three required adjunctive airway procedures. BTX doses ranged from 10 to 50 units, titrated progressively over multiple sessions based on response and tolerance, targeting interarytenoid, thyroarytenoid, and lateral cricoarytenoid muscles. Post-treatment laryngoscopy showed improved glottic opening in nine (90%) patients. Non-tracheostomized patients showed marked improvement in dyspnea and swallowing function. Transient mild voice changes occurred in three patients but resolved within 4-6 weeks, and no aspiration events were observed.

Conclusion

BTX demonstrates significant efficacy in pediatric VCD management, particularly in cases with neurologic etiology and severe symptoms refractory to conservative therapy. Objective measures support its role in improving glottic function and facilitating decannulation in appropriately selected patients.

## Introduction

Vocal cord dysfunction (VCD) is characterized by paradoxical adduction of the true vocal folds during inspiration due to dysregulated laryngeal adductor muscle activity, leading to intermittent inspiratory airway obstruction. Unlike vocal cord paralysis, which is fixed, VCD is a dynamic disorder with brief episodes of inappropriate inspiratory closure when the vocal folds should be opening. These episodes can cause significant breathing difficulty and, in severe cases, may become life-threatening. Pediatric VCD differs from the more common exercise-induced or psychogenic forms seen in adolescents and adults [1]. In younger children, VCD is often associated with underlying neurologic disorders, including brainstem abnormalities, neuromuscular disease, or impaired central control of laryngeal function [2]. Because symptoms are episodic and can vary in severity, management and diagnosis can be especially challenging.

Standard treatments include voice therapy, short-term use of anxiolytics during attacks, and tracheostomy in the most severe cases [3]. Management of pediatric VCD requires a multidisciplinary approach, typically involving pediatric otolaryngology, speech-language pathology, pulmonology, neurology, and behavioral health, particularly in medically complex or refractory cases. However, these approaches are often less effective in children with neurologically driven VCD. Botulinum toxin (BTX) has been considered as an alternative because it can weaken overactive laryngeal muscles while still allowing some function. Unlike surgical interventions, BTX is temporary and adjustable, which makes it useful for symptoms that fluctuate over time. By blocking acetylcholine release at the neuromuscular junction, BTX reduces excessive muscle activity for about three to six months. In VCD, this can help improve airflow during inspiration while still keeping enough tone for speech and swallowing. The temporary nature of BTX is particularly valuable in children, as it provides control during unstable periods but does not prevent recovery as the nervous system develops [4].

Despite these advantages, published evidence for BTX in pediatric VCD remains limited, with most reports consisting of single cases or small series. This study presents a dedicated case series of pediatric patients treated with BTX, emphasizing systematic patient selection, objective outcome measures, and detailed dosing strategies to help clarify its role as a treatment option in this challenging population.

## Materials and methods

Study design and patient selection

We conducted a retrospective chart review of all pediatric patients (age ≤18 years) diagnosed with VCD and treated with BTX at our tertiary pediatric ENT center between January 2021 and December 2024. Patients were included if they met the following criteria: (1) clinical diagnosis of VCD confirmed by laryngoscopy showing paradoxical vocal fold adduction during inspiration, (2) symptoms consistent with VCD such as inspiratory stridor, dysphagia, or dysphonia, (3) treatment with BTX injection, and (4) adequate follow-up data for outcome assessment. Diagnosis was established by direct laryngoscopy or flexible fiberoptic laryngoscopy. When feasible, exercise laryngoscopy was also performed to evaluate vocal fold motion under varying respiratory demands. Patients with static vocal fold paralysis, isolated posterior glottic stenosis, or other structural airway abnormalities were excluded unless VCD was present as a concurrent diagnosis. Institutional review board approval was obtained from the Oklahoma State University Center for Health Sciences (OSU-CHS) (approval number: 2025014), and the study adhered to ethical standards for retrospective pediatric research.

Patient characterization and comorbidity assessment

Detailed medical histories were obtained to identify potential risk factors for VCD. Special focus was placed on neurologic disorders, neuromuscular conditions, genetic syndromes, and prior airway procedures. For patients with tracheostomies, records were reviewed to determine whether the tracheostomy was placed primarily for VCD or for other airway issues. The decision to proceed with BTX therapy was based on the following: (1) severity of symptoms affecting breathing or quality of life, (2) lack of response to conservative management, including voice therapy when feasible, (3) neurologic comorbidities suggesting muscle hyperactivity as a likely mechanism, and (4) family understanding and agreement to pursue an off-label treatment.

Laryngoscopic evaluation

All patients underwent detailed laryngoscopic evaluation to document vocal fold position, motion, and paradoxical adduction. Diagnostic laryngoscopy was performed either under anesthesia with a direct approach or using awake flexible fiberoptic laryngoscopy when tolerated. VCD diagnosis was confirmed by demonstrating paradoxical vocal fold adduction during inspiration or by combining clinical findings with characteristic symptoms in patients too young or uncooperative for complete visualization. Follow-up laryngoscopies were performed after BTX treatment to assess vocal fold mobility, glottic opening, and changes in paradoxical movement. In cooperative patients, vocal fold motion was evaluated during variable respiratory efforts to better characterize treatment response.

At present, no validated pediatric VCD severity grading system exists. Therefore, laryngoscopic findings, including the degree of paradoxical adduction and glottic opening, as well as clinical symptoms such as stridor severity, respiratory distress, and swallowing function, were systematically evaluated to assess severity and monitor treatment response across patients.

Clinical symptom assessment

Swallowing function was assessed using fiberoptic endoscopic evaluation of swallowing (FEES) or modified barium swallow studies when feasible. Airway evaluation was performed through pre- and post-treatment laryngoscopy, including exercise laryngoscopy when tolerated, to assess glottic opening and paradoxical vocal fold motion (PVFM). For tracheostomized patients, decannulation status served as the primary outcome measure. Clinical responses were further categorized as complete resolution, significant improvement, moderate improvement, or minimal/no improvement based on stridor severity, feeding ability, and respiratory distress. Safety was monitored by clinical examination and caregiver reporting of adverse events, with particular attention to feeding or breathing difficulties following injection.

BTX injection technique

All BTX injections were performed by a fellowship-trained pediatric otolaryngologist under general anesthesia using direct suspension laryngoscopy for visualization, with injections delivered transorally under endoscopic guidance. Procedural images were not included due to the retrospective nature of the review and pediatric privacy considerations; however, key technical details are provided to support reproducibility. Target muscles included the interarytenoid, thyroarytenoid, and lateral cricoarytenoid muscles, alone or in combination, as these intrinsic laryngeal adductor muscles play a primary role in inappropriate inspiratory closure in VCD. BTX dosing was individualized based on patient size, symptom severity, and prior treatment response, with initial doses typically ranging from 0.5 to 1.0 units/kg divided across injection sites and titrated upward in subsequent sessions as clinically indicated. Injection targets were chosen based on paradoxical inspiratory adduction patterns on laryngoscopy, with the interarytenoid treated in all patients due to its role in posterior glottic closure, and the thyroarytenoid/lateral cricoarytenoid injected when medial or anterior fold hyperadduction was observed to optimize airway opening while preserving swallowing function.

Statistical analysis

Descriptive statistics were used to summarize patient demographics, comorbidities, treatment characteristics, and outcomes. Categorical variables are reported as numbers (%), and continuous variables as medians (ranges). Given the small sample size and retrospective nature of the study, no inferential statistical testing was performed.

## Results

Patient demographics and characteristics

The study cohort included 10 pediatric patients (six female patients and four male patients), ranging in age from 12 days to 17 years (median age: 2.8 years). Seven (70%) patients had notable comorbidities, including congenital heart disease (n = 2), chromosomal abnormalities (n = 2), neuromuscular disorders (n = 2), and DiGeorge syndrome (n = 1). Three patients had isolated VCD without additional underlying conditions. Four (40%) patients had tracheostomies at the time of their first BTX treatment, all placed specifically for VCD-related airway obstruction and severe stridor rather than other airway disease. The remaining six patients presented with significant but non-life-threatening symptoms that impaired quality of life or feeding. Detailed patient demographics, comorbidities, treatments, and outcomes are summarized in Table 1.

**Table 1:** Patient characteristics and outcomes BTX dose ranges reflect the lowest to highest per-session dose administered across the treatment course. “Number of sessions” indicates the total number of BTX treatments received. Transient improvement = benefit lasting <4 weeks; sustained improvement = benefit lasting ≥3 months. Ages are in years unless listed in days or months for infants. “Age” reflects age at first BTX session (not age at diagnosis or tracheostomy). All tracheostomies were placed prior to BTX for VCD-related airway obstruction (≤9 months to 4 years). For tracheostomized patients, BTX was administered before decannulation; Patient 1 received three sessions pre-decannulation and five maintenance sessions post-decannulation. BTX: botulinum toxin (onabotulinumtoxinA), VCD: vocal cord dysfunction

Patient	Sex	Age (years) (unless otherwise specified)	Significant comorbidities	Presentation	Tracheostomy	BTX dose per session (units)	BTX frequency	Outcome
1	Female	3	None	Stridor, dysphagia	Yes	12.5-50	8	Sustained improvement; decannulated after three sessions; five maintenance sessions post-decannulation
2	Male	2.5	None	Stridor, dyspnea	Yes	14-50	6	Sustained improvement; decannulated after six sessions
3	Female	4	DiGeorge	Stridor, dysphagia	Yes	12.5	3	Sustained improvement; decannulated after three sessions with tracheoplasty
4	Female	9 months	Congenital heart disease	Stridor	Yes	30	2	Sustained improvement; decannulated after two sessions
5	Male	5	Hypoxic brain injury	Stridor, dysphonia, dysphagia	No	12-25	5	Sustained improvement; breathing improved, oral feeds tolerated
6	Male	2	Congenital myasthenia gravis	Stridor, dysphagia	No	10-12	1	Sustained improvement; aspiration improved, intermittent stridor
7	Female	1	Chromosomal abnormality, epilepsy	Stridor, dysphagia, dysphonia	No	25	3	Sustained improvement; improved breathing, persistent dysphonia
8	Female	16	None	Stridor, dysphonia	No	25-50	5	Sustained improvement; breathing and voice improved
9	Male	12 days	X-linked myotubular myopathy	Stridor	No	12.5-30	3	Transient improvement in stridor (<4 weeks); no sustained benefit
10	Female	2	None	Stridor, dysphagia	No	25-50	5	Limited improvement, later required reconstruction

Clinical presentation and laryngoscopic findings

All 10 patients presented with inspiratory stridor as the primary complaint. Other symptoms included dysphagia (n = 6), dysphonia (n = 3), and feeding difficulties requiring gastrostomy tube placement (n = 3). Laryngoscopy confirmed paradoxical vocal fold adduction during inspiration in all patients. In younger children unable to tolerate awake procedures, diagnosis was established by examination under anesthesia, along with clinical features consistent with VCD. Findings varied in severity, ranging from complete inspiratory glottic closure to partial but clinically significant adduction that narrowed the airway. Importantly, all patients had normal vocal fold mobility during phonation when assessable, distinguishing VCD from static vocal cord paralysis.

BTX treatment details and dosing

BTX doses ranged from 10 to 50 units per session (median: 25 units), determined by patient size and symptom severity. The interarytenoid (n = 10), thyroarytenoid (n = 8), and lateral cricoarytenoid (n = 6) muscles were most frequently injected, usually bilaterally. Nearly all patients (9/10) underwent multiple treatment sessions, with intervals of 3-8 months depending on symptom recurrence. Initial doses were conservative (0.5-0.75 units/kg) and escalated in subsequent sessions if response was inadequate. Three patients required up to 1.5 units/kg to achieve optimal symptom relief, with no adverse events reported at these higher doses. All tracheostomies were placed prior to BTX initiation, and decannulation occurred after 2-6 BTX sessions, depending on symptom improvement, with three patients requiring adjunctive airway procedures. For all tracheostomized patients, BTX was administered before decannulation; maintenance BTX was used as needed after decannulation, including Patient 1 (five post-decannulation sessions).

Laryngoscopic response

Post-treatment laryngoscopy showed improved glottic opening in most patients. Nine of 10 (90%) patients demonstrated reduced hyperadduction and better airway patency following BTX injection. Responses varied, with some achieving near-normal inspiratory opening and others showing partial but meaningful improvement. One patient with X-linked myotubular myopathy showed minimal change, likely due to the complexity of the underlying neuromuscular disease. Follow-up laryngoscopies, typically performed 2-6 months post-injection, confirmed sustained benefit until BTX effects waned and repeat treatment was needed.

Clinical symptom response

All patients experienced symptomatic improvement after BTX treatment. Inspiratory stridor resolved completely in six patients and improved substantially in three others. Five of six patients with baseline dysphagia demonstrated feeding improvements, advancing to more normal intake. Follow-up laryngoscopy confirmed improved glottic opening in most patients, except in the case of X-linked myotubular myopathy, where minimal change was seen and only modest symptomatic relief in breathing was reported, consistent with the multifactorial nature of respiratory compromise in this condition.

Adverse events and safety profile

No major adverse events occurred. Three (30%) patients developed mild, temporary voice changes that resolved within 4-6 weeks without intervention. Careful monitoring for swallowing issues revealed no aspiration or new feeding compromise. No patient required emergency airway intervention. While concerns exist that BTX could worsen dysphagia, this series found stable or improved swallowing in patients with preexisting difficulties, likely due to precise injection technique and dosing targeted at abnormal adduction while preserving airway protection.

## Discussion

This case series demonstrates that botulinum toxin injection is an effective therapeutic option for pediatric vocal cord dysfunction, particularly in patients with neurologic comorbidities and refractory symptoms. Our findings align with emerging evidence supporting BTX use in carefully selected pediatric patients with paradoxical vocal fold motion.

Mechanism of action and treatment rationale

BTX induces temporary chemodenervation of laryngeal adductor muscles by blocking acetylcholine release at the neuromuscular junction [4]. This mechanism is particularly valuable when muscle hyperactivity or central dysregulation drives paradoxical glottic closure during inspiration. In pediatric VCD, excessive activation of intrinsic adductor muscles (interarytenoid, thyroarytenoid, and lateral cricoarytenoid) leads to inappropriate inspiratory closure; targeting these muscles reduces aberrant adduction while preserving airway protection. The reversible nature of BTX further supports its use in children, where ongoing neurologic development may influence disease trajectory over time [4].

Efficacy and predictors of response

Our series demonstrated a 90% laryngoscopic response rate, consistent with established efficacy data from adult populations. In adults with spasmodic dysphonia, randomized controlled trials have shown significant symptomatic improvement following BTX injection, while large retrospective series report response rates exceeding 85% [5,6]. Although pediatric data remain limited, published case reports describe comparable benefits. For instance, Cheng et al. (2014) reported successful management of paradoxical vocal fold motion in a child with cerebral palsy using periodic BTX injections, which avoided tracheostomy and improved both breathing and phonation [7].

Importantly, treatment response is not uniform and appears influenced by underlying neurologic etiology. In a large pediatric cohort, children with central nervous system disorders (e.g., hypoxic-ischemic injury and Arnold-Chiari malformation) had higher tracheostomy rates than those with idiopathic paralysis [8]. In our series, the patient with X-linked myotubular myopathy showed minimal sustained benefit, highlighting how progressive neuromuscular disease may limit BTX efficacy. Conversely, children with static neurologic injuries have shown more durable responses; for example, a child with cerebral palsy and PVFM achieved sustained symptom control and tracheostomy avoidance with periodic BTX injections [7]. This difference likely reflects underlying laryngeal neurophysiology (e.g., hyper-responsiveness and central control) rather than uniform drug effect, as reviewed by Dunn et al. [3]. Clinical effects of BTX in pediatric spasticity typically last 3-6 months, consistent with patterns observed in cerebral palsy cohorts, although substantial inter-individual variation occurs depending on disease severity and progression [9].

Another critical outcome domain is tracheostomy avoidance and decannulation. The largest pediatric experience to date comes from a 2008 series of seven children with bilateral vocal fold paralysis treated with botulinum toxin injection into the cricothyroid and strap muscles, in which six (85.7%) patients avoided tracheostomy [10]. A subsequent 2014 report described six pediatric cases treated with cricothyroid-only injection, with five (83.3%) averting tracheostomy and one successfully decannulated [11]. In our series, four tracheostomized VCD patients all ultimately achieved decannulation, although three required adjunctive surgical procedures. For a broader context, a 2024 systematic review of 320 pediatric bilateral vocal fold paralysis patients reported decannulation rates of 76.7% with conservative management and 62.5%-84.6% across various surgical interventions [12]. When viewed against these benchmarks, BTX appears to be a promising adjunctive tool within a multidisciplinary airway strategy. However, the cumulative pediatric literature remains limited, with fewer than 25 patients across all published series, precluding definitive conclusions about its role as a primary decannulation therapy.

Safety considerations

Safety data specific to pediatric laryngeal botulinum toxin applications remain limited. Broader pediatric experience from spasticity management indicates systemic adverse events in approximately 3.6% of injection episodes, with preexisting dysphagia or aspiration pneumonia conferring the highest risk (odds ratio (OR): 3.4 and 2.3, respectively) [13]. Expert consensus guidelines emphasize that although uncommon, serious adverse events, including respiratory failure due to toxin diffusion, have been reported after injections near anatomically vulnerable regions such as the larynx and pharynx [14]. In our series, no major complications occurred. Three patients experienced transient voice changes resolving within 4-6 weeks, and no aspiration events were observed. This favorable profile likely reflects conservative dosing, precise injection technique, and close post-procedural monitoring. Nonetheless, the potential for severe complications underscores the importance of thorough informed consent discussions and vigilant clinical surveillance, particularly given the paucity of pediatric-specific safety data for this indication.

## Conclusions

Botulinum toxin injection represents a valuable therapeutic option for pediatric patients with vocal cord dysfunction (VCD), particularly those with neurologic etiologies and severe, refractory symptoms. This case series demonstrates that BTX can improve vocal fold function, facilitate tracheostomy decannulation, and enhance quality of life in carefully selected patients. Its favorable safety profile, reversibility, and non-surgical nature make it especially appealing in children, where permanent airway procedures carry significant long-term risks. Optimal outcomes, however, depend on thoughtful patient selection, systematic outcome assessment, and individualized dosing strategies that account for etiology and disease trajectory. While BTX is not a universal first-line therapy, it should be recognized as a meaningful adjunct for complex or refractory VCD. Future prospective, multicenter studies with standardized protocols are essential to clarify its precise role, refine dosing guidelines, and integrate BTX more fully into comprehensive pediatric airway management.
